# Arabidopsis PCaP2 Plays an Important Role in Chilling Tolerance and ABA Response by Activating CBF- and SnRK2-Mediated Transcriptional Regulatory Network

**DOI:** 10.3389/fpls.2018.00215

**Published:** 2018-03-08

**Authors:** Xianling Wang, Lu Wang, Yu Wang, Huan Liu, Dan Hu, Ning Zhang, Shaobin Zhang, Huiying Cao, Qijiang Cao, Zhihong Zhang, Shuang Tang, Dandan Song, Che Wang

**Affiliations:** ^1^College of Bioscience and Biotechnology, Shenyang Agricultural University, Shenyang, China; ^2^Department of Medicine, HE University School of Clinical Medicine, Shenyang, China; ^3^Luoyang High-Tech Zone No. 2 Experimental School, Henan, China

**Keywords:** PCaP2, chilling, CBF, ABA, SnRK2, Arabidopsis

## Abstract

Chilling stress affects plant growth and productivity. However, the multi-underlying mechanisms of chilling tolerance are not well understood. Arabidopsis PCaP2 is involved in regulating the dynamic of microtubules (MTs) and F-actin and Ca^2+^-binding ability. Here, the results showed that the *PCaP2* expression was highly induced in roots, cotyledons, true leaves, lateral roots and flowers under cold stress. Compared with the wild type, *PCaP2*-overexpressing plants displayed the enhanced tolerance, whereas its RNAi and mutant were more sensitive in seed germination, seedling and reproductive growth under chilling stress in Arabidopsis. In addition, PCaP2 was also a positive regulator of ABA signaling pathway by analyzing the expression of *PCaP2* and the phenotypes of PCaP2-overexpressing, mutant and RNAi plants under ABA treatment. Interestingly, disruption of *PCaP2* inhibited the expression of *CBF1*, *-3* and CBF-target *COR* genes, while increased the *CBF2* expression in response to cold or ABA. Moreover, we found that SnRK2s were involved in cold stress and *PCaP2* mutants down-regulated the transcription level of SnRK2.2, -2.3 and SnRK2-mediated downstream genes including *ABF2*, *RD29A*, *KIN1*, *KIN2*, but up-regulated *SnRK2.6*, *ABF1*, *-3*, *-4* in ABA and cold treatments. It is well-accepted that PCaP2 as a Ca^2+^-binding protein triggers the gene expression to enhance plant chilling tolerance. Our further studies showed that MT destabilizing activity of PCaP2, but not F-actin-severing function, may be involved in chilling stress. Taken together, our results highlight that PCaP2 plays an important role in chilling tolerance and ABA response by triggering the CBF- and SnRK2-meditated transcriptional regulatory pathways, providing novel evidences of underlying mechanisms of multi-pathways in chilling stress.

## Introduction

Cold stress, including chilling (0–20°C) and freezing (<0°C) temperature, severely affecting plant growth, development and agricultural productivity. Plant cells can sense cold stress through low temperature-induced change of membrane fluidity. Subsequently, many signals such as Ca^2+^ signal, ROS, ABA, SA and other phytohormones can affect the expression pattern of various genes, such as protein kinase genes, transcription factor genes and *COR* genes, and physiological activities (Chinnusamy et al., 2007).

C-Repeat Binding Factor/DRE Binding Factor1 (CBF/DREB1)-*COR* signaling pathway is one of the most well understood transcriptional regulation pathways in plant response to cold stress. In *Arabidopsis thaliana*, CBFs including CBF1/DREB1b, CBF2/DREB1c, CBF3/DREB1a, CBF4/DREB1d, and CBF5 (Stockinger et al., 1997; Gilmour et al., 1998; Jaglo-Ottosen et al., 1998; Liu et al., 1998; Medina et al., 1999; Haake et al., 2002; Novillo et al., 2004; Lermontova et al., 2006) can induce the expression of *COR* genes, such as *COR15A*, *COR15B*, *RD29A*, *RD29B*, *KIN1*, and *KIN2* (Novillo et al., 2007), to increase plant cold tolerance. CBF homologs have been cloned from both cold-tolerant (wheat, barley and grape) and cold-sensitive (rice, maize, tomato, and cherry) crops (Anderson et al., 1994; Sangwan et al., 2001; Hsieh et al., 2002; Vannini et al., 2004; Mastrangelo et al., 2005; Oh et al., 2005). Recently, two different results of the chilling inducible phenotype of *cbf1 cbf2 cbf3* triple mutants have been reported in Arabidopsis. Jia et al. (2016) found the tolerance phenotype of *cbf* triple mutants is enhanced in response to chilling, while Zhao et al. (2016) results showed that there is no significant phenotype of *cbf* triple mutants in chilling stress. In addition, more reports have been proved that the overexpression plants of Arabidopsis *CBF*s or other species *CBF*s the overexpressing plants confer chilling tolerance (Hsieh et al., 2002; Yun et al., 2010; Huang et al., 2012; An et al., 2016, 2017; Cheng et al., 2016), illustrating the important role of CBFs and the complex mechanism of CBFs in chilling stress.

Abscisic acid, as an important signal molecule, can mediates abiotic stress (drought, salt, and cold stress) signal transduction and tolerance. It has been found that ABA activates protein kinases such as CPK4, CPK11, SnRK2s, and OST1, then these induced protein kinases stimulate the expression of ABA-dependent transcription factors, such as ABIs and ABFs binding to the ABA responsive *cis*-regulatory elements in the promoter region of abiotic stress-responsive genes (Trivedi et al., 2016). Approximately 10% of ABA-responsive genes are involved in response to cold stress (Kreps et al., 2002), and some of these genes or proteins have been studied in more details. For example, ectopic expression *ABI3* confers ability to express *COR* genes in vegetative tissues and enhances cold tolerance in Arabidopsis (Tamminen et al., 2001). Overexpression of *CBF4/DREB1d* mediated by ABA enhances drought and cold tolerances in Arabidopsis (Haake et al., 2002). Cassava *MeCBF1* significantly induced by salt, PEG and ABA treatment and enhances chilling tolerance when overexpressed in Arabidopsis and cassava (An et al., 2017). Although limited investigations of the molecular mechanisms of the ABA-mediated cold signaling pathway have been conducted, some results indicate a crosstalk between ABA- and CBF-pathway in cold stress.

Arabidopsis PCaP2 is widely expressed in the roots, hypocotyls, and cotyledons and highly expressed in root hairs and pollen tubes by GUS activity analysis (Wang X. et al., 2007; Kato et al., 2010a). Previous studies indicate that PCaP2 is involved in three main aspects. Firstly, PCaP2 regulates directional cell growth and cortical MT organization by destabilizing MTs (Wang X. et al., 2007). Secondly, PCaP2 exhibits Ca^2+^-dependent filamentous (F)-actin severing activity *in vitro*, and this function involves in guiding the direction of pollen tube growth (Zhu et al., 2013). Lastly, PCaP2 is a Ca^2+^-binding protein involved in regulating the growth of root hairs and its mRNA expression level is induced by ABA, GA3, SA, heat, cold, drought, and osmotic stress (Kato et al., 2010a, 2013), suggesting it may function as a crosstalk factor in response to abiotic stress and phytohormone signals. One significant result is the mRNA level of *PCaP2* is increased about eightfold by cold treatments (Kato et al., 2010a), however, the more evidences of PCaP2 are still unknown in cold stress.

In this study, we found that the expression of *PCaP2* was induced in roots, cotyledons, true leaves, lateral roots and flowers under low temperature treatments. Next we tested the phenotypes of wild-type (WT), *PCaP2*-OE, *PCaP2*-RNAi and mutants, including the seed germination rates, leaf area, primary roots, lateral roots, flowers and siliques under chilling treatments. The results showed that *PCaP2*-OE lines showed the chilling tolerance phenotype and *PCaP2*-RNAi and mutant lines chilling hyper-sensitivity phenotype. In addition, *PCaP2* was also induced by ABA and ABA-inducible phenotype of *PCaP2*-OE, *PCaP2*-RNAi and mutant lines were similar with that in chilling treatments. Further analysis found that PCaP2 positively regulated the expression of *CBF1*, *CBF3*, CBF-target genes, *SnRK2.2*, -*2.3* and *ABF2* (SnRK2-mediated gene) and negatively mediated the expression of *CBF2* in cold or ABA treatments. Taken together, our results highlight that *PCaP2* plays an important and positive function in chilling tolerance and ABA response and it can trigger CBF-and SnRK2-transcriptional network, providing some novel evidences of underlying mechanisms of multi-pathways in chilling stress.

## Results

### The Expression of *PCaP2* Is Highly Induced in All Tissues Under Chilling Stress

**FIGURE 1:**
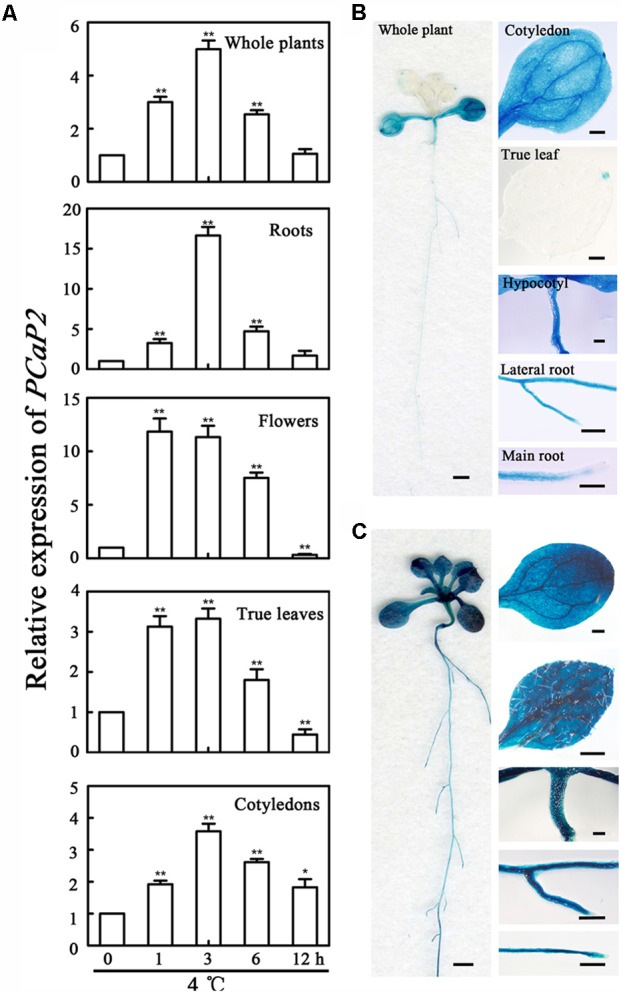
Expression pattern of *PCaP2* under cold stress. **(A)** The relative expression of *PCaP2* in different parts of the 14-day-old plants with exposure to 4°C at 0, 1, 3, 6, and 12 h was determined by qRT-PCR assay. Values are the mean ± SD (*n* = 3, Student’s *t*-test, control vs. treatment by genotype, ^∗^*P* < 0.05, ^∗∗^*P* < 0.01). **(B,C)** The expression pattern of *PCaP2* was revealed by GUS staining of p*PCaP2*::GUS transgenic plants in normal (22°C, the same below) **(B)** and 4°C for 3 h **(C)** conditions. The scale bar is 1 mm in the pictures of whole plant. The scale bar is 0.25 mm in the pictures of cotyledon, true leaf, hypocotyl, lateral root and main root.

It has been reported that the expression of *PCaP2* in whole seedlings is significantly induced after treated by 4°C for 6 h (Kato et al., 2010a). To fully understand the expression pattern of *PCaP2* under cold stress, the expression was detected in more details under 4°C treatments by analysis of qRT-PCR and GUS staining. The results showed that the expression of *PCaP2* was induced in roots, cotyledons, true leaves, flowers and the whole seedlings by 4°C treatments from 1 to 12 h by qRT-PCR assay, with the peak level of the increased 16.64 ± 1.06-folds at 3 h after 4°C treatments (**Figure 1A**). The highest cold-induced expression of *PCaP2* was detected in roots, compared with that in other tissues, (**Figure 1A**). To confirm the result of qRT-PCR assay, the promoter activity was detected using GUS as a reporter treated by 4°C for 3 h. The results showed that GUS activity was greatly induced in the whole seedlings, cotyledons, true leaves, hypocotyls, primary roots and lateral roots after 3 h treatment by 4°C, which is consistent with the above results from qRT-PCR (**Figures 1B,C**).

### *PCaP2*-OE Displays the Increased Tolerance While Its RNAi and Mutant Seedlings Are Hypersensitive in Response to Chilling Stress

**FIGURE 2:**
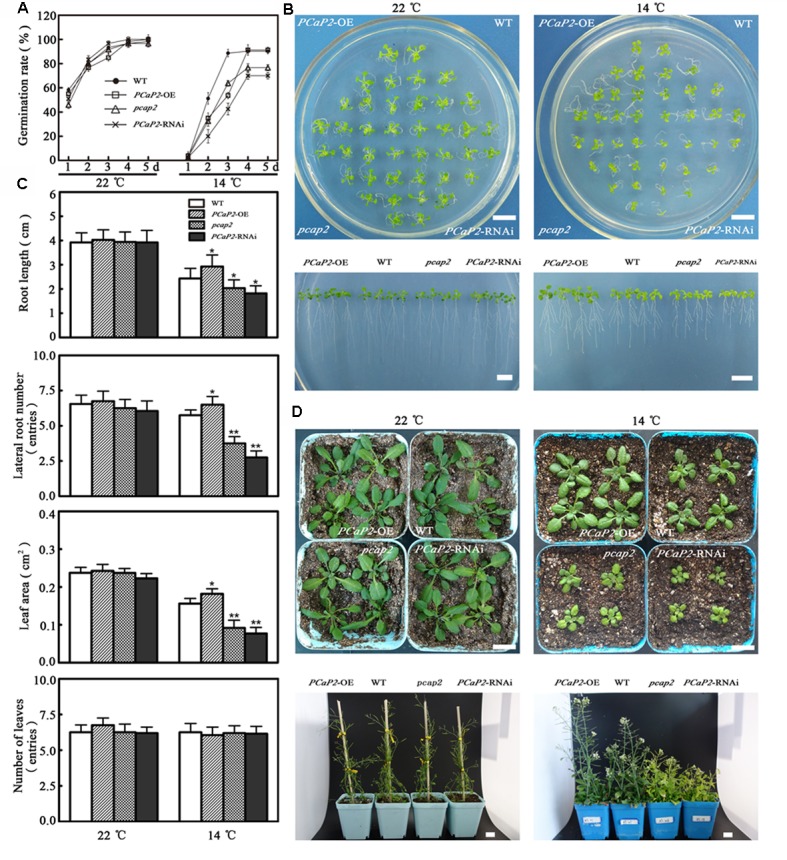
Effect of PCaP2 on Arabidopsis seed germination, seedling and reproduction growth under chilling stress. **(A)** The seed germination rates were recorded in 1/2 MS media at normal and low temperature (14°C, the same below) during a period from 1 to 5 days after stratification for the WT, *PCaP2*-OE, *pcap2*, and *PCaP2*-RNAi (*n* = 3, Student’s *t*-test, genotypes vs. WT in same condition, ^∗^*P* < 0.05, ^∗∗^*P* < 0.01). **(B)** Arabidopsis seeds of *PCaP2*-OE, WT, *pcap2*, and *PCaP2*-RNAi were sown on 1/2 MS medium at 22°C for 4 days, then those 4-day-old seedlings transferred to 14°C conditions for 11 days. Scale bar = 1 cm. **(C)** Root length, the number of lateral roots and leaves, and leaf area of WT, *PCaP2*-OE, *pcap2*, and *PCaP2*-RNAi were examined after growth at normal and low temperature at the indicated times (*n* = 3, Student’s *t*-test, genotypes vs. WT in same condition, ^∗^*P* < 0.05, ^∗∗^*P* < 0.01). **(D)** The seedling growth of *PCaP2*-OE, WT, *pcap2*, and *PCaP2*-RNAi in matrix soil at normal and low temperature for 40 and 60 days, respectively. Scale bar = 1 cm.

To analyze the function of PCaP2 in plant chilling tolerance, the phenotypes of a *PCaP2*-OE line (Supplementary Figure S1), a knockout of *PCaP2*-RNAi line (Wang X. et al., 2007; Supplementary Figure S1) and a knockdown of T-DNA insertion (*pcap2*) line (SALK_021652) (Kato et al., 2013; Zhu et al., 2013; Supplementary Figure S1) were investigated under normal (22°C) and 14°C conditions. Under normal conditions, the germination rates of *PCaP2*-OE, *PCaP2*-RNAi and *pcap2* were slightly different than these of WT seeds from 1 to 3 days, but the germination of these seeds was identical at 4 and 5 days (**Figure 2A**). However, chilling significantly inhibited the germination of all these seeds, the germination rates of *PCaP2*-OE, *PCaP2*-RNAi and *pcap2* were obviously different than these of WT seeds at 2 days, then the germination rates of WT and *PCaP2*-OE were similar, and they were higher than these of *PCaP2*-RNAi and *pcap2* with chilling treatments at 4 and 5 days (**Figure 2A**). When the 4-day-old seedlings grown for 11 days in 1/2MS, the grown status had no significant difference among WT, *PCaP2*-OE, *PCaP2*-RNAi, and *pcap2* lines under normal conditions, however, *PCaP2*-OE seedlings had longer roots, larger leaves and lateral roots than WT under 14°C, while *PCaP2*-RNAi and *pcap2* had the opposite situation (**Figures 2B,C**). The similarly different growth of these seedlings was found when 7-day-old seedlings were transferred to matrix soil at 22°C for 40 days (the top of **Figure 2D**) or 14°C for 60 days (the bottom of **Figure 2D**). In the process of reproductive growth, *PCaP2*-OE, *PCaP2*-RNAi, and *pcap2* were identical under normal conditions, while *PCaP2*-OE seedlings showed stronger, more flowers, and more siliques than WT, while *PCaP2*-RNAi and *pcap2* had the opposite situation (**Figure 2D**). Compared with the chilling-inducible phenotype of *pcap2*, the more obvious features of *PCaP2*-RNAi may result from the different expression of *PCaP2* between *pcap2* and *PCaP2*-RNAi (**Figure 2D**). Our results suggest that PCaP2 positively regulates the chilling tolerance of Arabidopsis in seed germination, seedling and reproductive growth.

### ABA Induces the Expression of *PCaP2*

**FIGURE 3:**
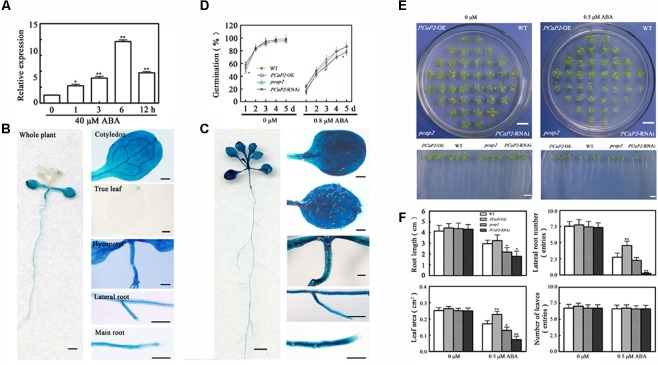
Expression pattern of *PCaP2* and effect of PCaP2 on Arabidopsis seed germination and seedling growth with exogenous ABA treatments. **(A)** The relative expression of *PCaP2* of the 14-day-old plants with 40 μM ABA treatment for 0, 1, 3, 6, and 12 h was determined by qRT-PCR assay. Values are the mean ± SD (*n* = 3, Student’s *t*-test, genotypes vs. WT in same condition, ^∗^*P* < 0.05, ^∗∗^*P* < 0.01). **(B,C)** The expression pattern of *PCaP2* was revealed by GUS staining of p*PCaP2*::GUS transgenic plants in normal (without ABA treatment) **(B)** and 40 μM ABA treatment. **(C)** The scale bar is 1 mm in the pictures of whole plant. The scale bar is 0.25 mm in the pictures of cotyledon, true leaf, hypocotyl, lateral root and main root. **(D)** The seed germination rates were recorded in 1/2 MS media supplemented with 0 and 0.8 μM ABA during a period from 1 to 5 days after stratification for the WT, *PCaP2*-OE, *pcap2*, and *PCaP2*-RNAi (*n* = 3, Student’s *t*-test, genotypes vs. WT in same condition, ^∗^*P* < 0.05, ^∗∗^*P* < 0.01). **(E)** Arabidopsis seeds of *PCaP2*-OE, WT, *pcap2*, and *PCaP2*-RNAi were sown on 1/2 MS medium, then those 4-day-old seedlings transferred to 1/2 MS medium supplemented with 0 and 0.5 μM ABA for 13 days. Scale bar = 1 cm. **(F)** Root length, the number of lateral roots and leaves, and leaf area of WT, *PCaP2*-OE, *pcap2*, and *PCaP2*-RNAi were examined after growth on 1/2 MS medium supplemented with 0 and 0.5 μM ABA at the indicated times (*n* = 3, Student’s *t*-test, genotypes vs. WT in same condition, ^∗^*P* < 0.05, ^∗∗^*P* < 0.01).

Abscisic acid is another regulatory factor in chilling stress, and the higher expression level of *PCaP2* has been found in ABA treatments (Kato et al., 2010a; Trivedi et al., 2016). To investigate whether PCaP2 is also regulated by ABA signaling in chilling stress, the expression of *PCaP2* was firstly examined in exogenous ABA treatments. qRT-PCR and GUS staining showed that the level of *PCaP2* mRNA was markedly increased with ABA treatments from 1 to 12 h and the expression of *PCaP2* was induced in the whole seedlings, cotyledons, true leaves, hypocotyls, primary roots and lateral roots after 12 h treatment by 40 μM ABA (**Figures 3A–C**).

### *PCaP2*-OE Displays the Increased Tolerance, While Its RNAi and Mutant Are Hypersensitive, in Exogenous ABA Treatments

Then we examined the seed germination rates and seedling growth of WT, *PCaP2*-OE, *PCaP2*-RNAi and *pcap2* under 0 or 0.8 μM ABA treatments. The results showed that there were no significant differences observed in seed germination rates without ABA treatments (**Figure 3D**). However, the germination rates of WT and *PCaP2*-OE were similar, and they were higher than these of *PCaP2*-RNAi and *pcap2* with exogenous ABA treatments (**Figure 3D**). The growth status of WT, *PCaP2*-OE, *PCaP2*-RNAi, and *pcap2* were identical under control conditions (**Figure 3E**). However, under ABA treatments, *PCaP2*-OE seedlings had larger leaf area, longer primary roots, and more lateral roots than WT seedlings, while *PCaP2*-RNAi and *pcap2* had opposite phenotypes, which is consistent with the chilling-inducible phenotypes (**Figure 3F**). These results suggest that PCaP2 acts as a positive factor in ABA signaling pathway and the involvement of PCaP2 in chilling might be mediated by ABA signaling pathway.

### PCaP2 Affects the Expression of *CBF*s and CBF-Target *COR* Genes With Low Temperature and Exogenous ABA Treatments

**FIGURE 4:**
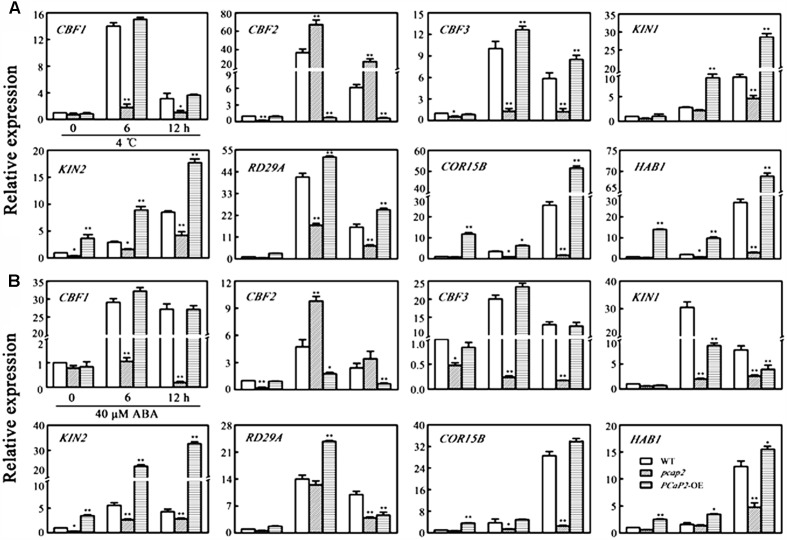
Effect of PCaP2 on the expression of *CBF*s and CBF-target *COR* genes with low temperature and exogenous ABA treatments. **(A)** The relative expression of *CBF*s and CBF-target *COR* genes of the 14-day-old plants with exposure to 4°C for 0, 6, and 12 h was determined in WT, *pcap2*, and *PCaP2*-OE by qRT-PCR assay. **(B)** The relative expression of *CBF*s and CBF-target *COR* genes of the 14-day-old plants with 40 μM ABA treatment for 0, 6, and 12 h was determined in WT, *pcap2*, and *PCaP2*-OE by qRT-PCR assay. Values are the mean ± SD (*n* = 3, Student’s *t*-test, genotypes vs. WT in same condition, ^∗^*P* < 0.05, ^∗∗^*P* < 0.01).

C-repeat binding Factor-signaling pathway is crucial to plants in response to cold signaling. To obtain clues as to whether PCaP2 plays a role in regulating *CBF*s and *COR* gene expression, we examined the expression of *CBF*s and CBF-target *COR* genes including *CBF1*, *CBF2*, *CBF3*, *COR15B*, *KIN1*, *KIN2*, *RD29A*, and *HAB1* (Novillo et al., 2004) in WT, *PCaP2*-OE and *pcap2* seedlings with 4°C treatments at 0, 6, and 12 h by qRT-PCR analysis. Previous studies have used the T-DNA mutant of *PCaP2* to analyze the function of PCaP2 (Kato et al., 2013; Zhu et al., 2013). Thus, the T-DNA mutant of *PCaP2* was chosen in the analysis of the gene expression in the present study. As shown in **Figure 4**, the expression level of all chosen genes, especially *CBF2*, *CBF3*, *KIN1*, *RD29A*, *KIN2*, was significantly decreased in *pcap2*, compared with that in WT under normal conditions. Under cold stress, the expression of all these genes, except for *CBF2*, was significantly inhibited in *pcap2* and increased in *PCaP2*-OE seedlings (**Figure 4A**). The results demonstrate that PCaP2 affects the expression of CBF-*COR* pathway genes and might enhance chilling tolerance by triggering *CBF1*, -*3* expression and inhibiting *CBF2* expression.

To further dissect whether PCaP2-mediated CBF-*COR* pathway genes was also involved in ABA signaling pathway, we examined the expression level of the above genes in WT, *PCaP2*-OE and *pcap2* seedlings with ABA treatments (**Figure 4B**). The results showed that only *CBF2* expression was significantly up-regulated and other gene expression was significantly down-regulated in *pcap2*, which is consistent with the above results in cold stress.

### PCaP2 Changes the Expression of SnRK2 Genes and SnRK2-Mediated ABA-Responsive Genes in Exogenous ABA and Low Temperature Treatments

**FIGURE 5:**
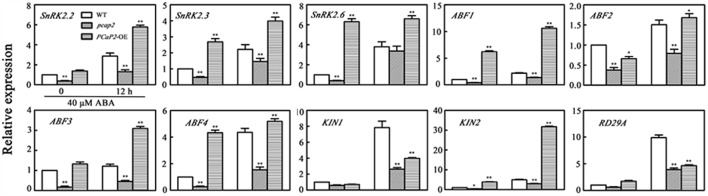
Effect of PCaP2 on the expression of *SnRK2* genes and SnRK2-mediated ABA-responsive genes with exogenous ABA treatments. The relative expression of ABA-inducible genes of the 14-day-old plants with 40 μM ABA treatment for 0 and 12 h was determined in WT, *pcap2*, and *PCaP2*-OE by qRT-PCR assay. Values are the mean ± SD (*n* = 3, Student’s *t*-test, genotypes vs. WT in same condition, ^∗^*P* < 0.05, ^∗∗^*P* < 0.01).

SnRK2s are a center of ABA signaling pathway and regulate the expression of *ABF*s, *ABI*s and some downstream ABA-responsive genes (Fujii et al., 2007; Kulik et al., 2011). To dissert how PCaP2 regulates the molecular pathway of ABA signal, we examined the expression of the following SnRK2 genes and SnRK2-mediated ABA-responsive genes in WT, *PCaP2*-OE and *pcap2* seedlings: *SnRK2.2*, *SnRK2.3* and *SnRK2.6* (Boudsocq et al., 2004); *ABF* genes (*ABF2/AREB1*, *ABF3* and *ABF4*) (Choi et al., 2000; Uno et al., 2000); *RD29A* (Yamaguchi-Shinozaki and Shinozaki, 1994); *KIN1* and *KIN2* (Kurkela and Borg-Franck, 1992). In the absence of exogenous ABA, compared with WT, the expression of all these genes decreased in *pcap2* seedlings. The expression of *SnRK2*s decreased between 2.5- and 3-fold, while *ABF*s decreased between 2.5 and 6.25-fold (**Figure 5**). In the presence of exogenous ABA, the mutation of *PCaP2* still inhibited the expression of *SnRK2s*, *ABF*s, *KIN1*, *KIN2*, and *RD29A* (**Figure 5**). On basis of the above results, PCaP2 is a positive factor in ABA signaling pathway, illustrating that disruption of *PCaP2* may play an inhibition role in the expression of some ABA-induced genes. Additionally, overexpression of *PCaP2* induced the expression of *SnRK2*s, *ABF*s, and *KIN2*. Thus, we speculate that PCaP2 can regulate the expression of *SnRK2*s and *ABF*s positively to participate in ABA signaling.

**FIGURE 6:**
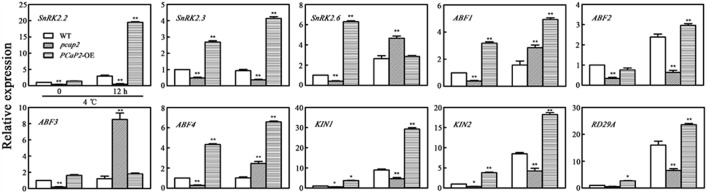
Effect of PCaP2 on the expression of *SnRK2* genes and SnRK2-mediated ABA-responsive genes under cold stress. The relative expression of ABA-inducible genes of the 14-day-old plants with exposure to 4°C for 0 and 12 h was determined in WT, *pcap2*, and *PCaP2*-OE by qRT-PCR assay. Values are the mean ± SD (*n* = 3, Student’s *t*-test, genotypes vs. WT in same condition, ^∗^*P* < 0.05, ^∗∗^*P* < 0.01).

To further dissect whether the above PCaP2-mediated ABA-responsive genes was also involved in low temperature stress, we examined the expression level of the above genes in WT, *PCaP2*-OE and *pcap2* seedlings under normal and low temperature conditions. In normal conditions, the expression of all these genes was declined in *pcap2* seedlings, compared with WT (**Figure 6**). In low temperature conditions, *pcap2* still decreased the expression of *SnRK2.2*, -*2.3*, *ABF2*, *KIN1*, *KIN2*, and *RD29A*, while induced the expression of *SnRK2.6*, *ABF1*, -*3*, -*4* (**Figure 6**), at the same time, *PCaP2*-OE showed the contrary effect on these gene expression. As shown in the above results, in response to cold stress, PCaP2 involves in chilling tolerance and ABA signaling pathway by regulating the expression of *SnRK2.2*, -*2.3*, *ABF2*, *KIN1*, *KIN2*, and *RD29A*. While these results are consistent with PCaP2 positively regulating *SnRK2.2*, *-2.3*, *ABF2*, *KIN1*, *KIN2*, and *RD29A*, the effects on *ABF1*, *-3*, *-4*, and *SnRK2.6* are less straightforward.

### MT Destabilizing Activity of PCaP2 May Be Involved in Plant Chilling Tolerance

**FIGURE 7:**
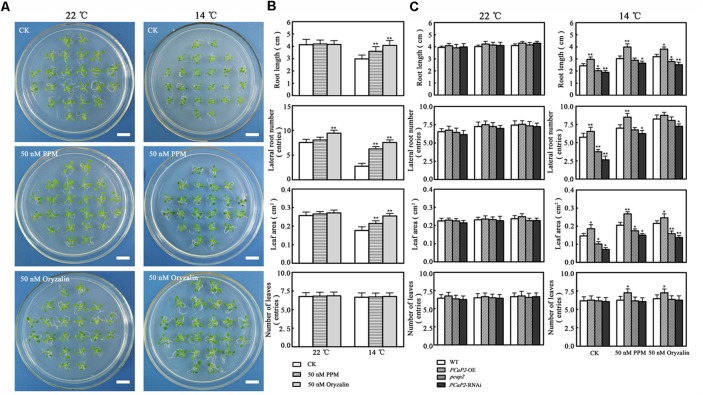
Effect of microtubule destabilizing activity of PCaP2 on Arabidopsis seedling growth under chilling stress. **(A)** Arabidopsis seeds of WT were sown on 1/2 MS medium at 22°C for 4 days, then the 4-day-old seedlings transferred to 1/2 MS medium supplemented without or with 50 nM oryzalin or 50 nM PPM for 16 days. Scale bar = 1 cm. **(B)** Root length, the number of lateral roots and leaves, and leaf area of WT were examined after growth at normal 50 nM oryzalin and 50 nM PPM at the indicated times (*n* = 3, Student’s *t*-test, genotypes vs. WT in same condition, ^∗^*P* < 0.05, ^∗∗^*P* < 0.01). **(C)** Arabidopsis seeds of WT, *PCaP2*-OE, *pcap2*, and *PCaP2*-RNAi were sown on 1/2 MS medium at 22°C for 4 days, then the 4-day-old seedlings transferred to 1/2 MS medium supplemented without or with 50 nM oryzalin or 50 nM PPM for 16 days. Root length, the number of lateral roots and leaves, and leaf area of WT, *PCaP2*-OE, *pcap2* and *PCaP2*-RNAi were examined after growth at normal 50 nM oryzalin and 50 nM PPM at normal (22°C) and low temperature (14°C) at the indicated times (*n* = 3, Student’s *t*-test, genotypes vs. WT in same condition, ^∗^*P* < 0.05, ^∗∗^*P* < 0.01).

To investigate whether MT destabilizing activity of PCaP2 is involved in response to chilling stress, we firstly analyzed whether MT destabilization in WT benefits to plant tolerance chilling stress. Thus, we monitored the phenotype of WT in the presence of MT-destabilizing drugs (oryzalin and PPM) in normal or chilling conditions. Compared with the no drug treatments, the growth of WT seedlings treated with 50 nM oryzalin or 50 nM PPM was not different in normal conditions (**Figure 7A**). However, in 14°C conditions, the seedlings with oryzalin or PPM treatments were longer roots, larger leaves, more numbers of leaves and lateral roots than that without drug treatments, which is consistent with chilling-inducible phenotype of *PCaP2*-OE seedlings, suggesting that the low-dose MT destabilizing drug benefits to plant chilling tolerance and chilling-inducible phenotype of *PCaP2*-OE might be obtained from MT destabilizing effects (**Figure 7B**).

To confirm the above results, we secondly monitored whether the phenotypes of *PCaP2*-RNAi and *pcap2* can be compensated by oryzalin and PPM in chilling conditions. The study found that the phenotypes of *PCaP2*-RNAi and *pcap2* treated by oryzalin or PPM were well growth, similarly with that of WT, in 14°C, illustrating the MT destabilization can partially compensate functional disruption of *PCaP2* in chilling stress (**Figure 7C**). These results suggest that MT destabilizing activity of PCaP2 may be involved in plant chilling tolerance.

**FIGURE 8:**
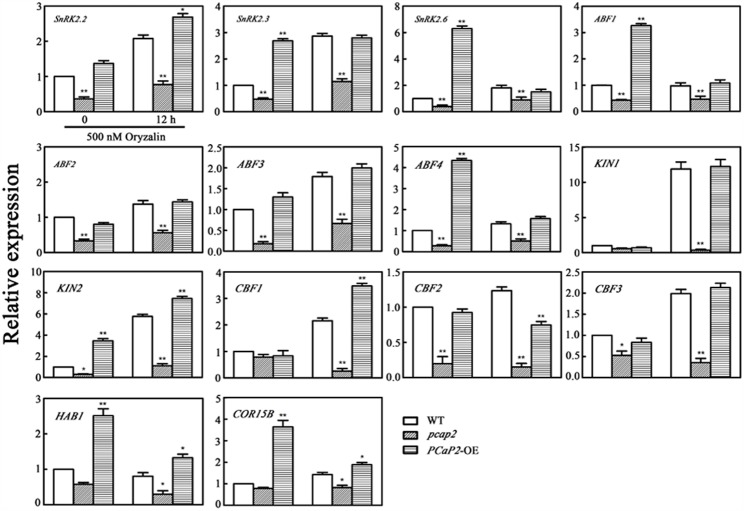
Effect of PCaP2 on the expression of *SnRK2* genes, SnRK2-mediated ABA-responsive genes, *CBF*s and CBF-target *COR* genes with oryzalin treatments. The relative expression of *SnRK2* genes, SnRK2-mediated ABA-responsive genes, *CBF*s and CBF-target *COR* genes of the 14-day-old plants with 500 nM oryzalin treatment for 0 and 12 h was determined in WT, *pcap2*, and *PCaP2*-OE by qRT-PCR assay. Values are the mean ± SD (*n* = 3, Student’s *t*-test, genotypes vs. WT in same condition, ^∗^*P* < 0.05, ^∗∗^*P* < 0.01).

To investigate whether MT destabilization effect on expression level of *CBF*s, CBF-target *COR* genes, *SnRK2* and *SnRK2*-mediated ABA responsive genes. We further examined the expression change of the above cold- and ABA-responsive genes in WT, *PCaP2*-OE and *pcap2* seedlings with oryzalin treatments under 22°C. The results showed that oryzalin treatments inhibited all these target genes expression in *pcap2* and increased these genes expression in *PCaP2*-OE seedlings (**Figure 8**). Moreover, we found that the increasing expression of *PCaP2*-OE seedlings in oryzalin treatments was not as strong as cold and ABA treatments (**Figure 8**), suggesting MT destabilization might be a partial role in inducing the expression of ABA-responsive genes, *CBF*s and CBF-target *COR* genes.

### F-Actin-Severing Activity of PCaP2 Is Not Involved in Response to Chilling Stress in Seedling Growth

We then asked whether F-actin-severing activity of PCaP2 is responsive to chilling stress. We treated *m1*, *m2* (expressing PCaP2 mutant proteins M1 and M2 specifically lacking F-actin-severing activity, respectively, in a *pcap2* background) and the *m7* (expressing a PCaP2 mutant protein M7 that retained F-actin-severing activity in a *pcap2* background) seedlings (the MT activity of PCaP2 isn’t effected in *m1*, *m2*, and *m7*) (Zhu et al., 2013), *PCaP2*-RNAi as a positive control, and WT with exposure to 14°C. The results showed no obvious differences among the WT, *m1*, *m2*, and *m7* under normal and chilling conditions (Supplementary Figure S2), suggesting that the F-actin-severing activity of PCaP2 could not participate in response to chilling stress in seedling growth.

## Discussion

It has been reported that PCaP2 is involved in the organization of cortical MTs and F-actin, and the binding-Ca^2+^ ability (Wang X. et al., 2007; Kato et al., 2010a, 2013; Zhu et al., 2013), and the transcription level of *PCaP2* can be induced by abiotic stress and some phytohormones, especially, cold-induced *PCaP2* expression with increased eightfold is the highest in all these treatments (Kato et al., 2010a), however, the mechanism of PCaP2 remains unknown in abiotic stress or phytohormone signaling pathways. Our results support a role for PCaP2 in Arabidopsis chilling tolerance and ABA response associated with activation of CBF- and SnRK2-mediated pathways.

### PCaP2 Plays an Important Role Under Chilling Stress

Previous data showed that the mRNA level of *PCaP2* in the whole plant is increased with 4°C treatments and *PCaP2* is mainly expressed in roots and flower tissues in normal condition (Wang X. et al., 2007; Kato et al., 2010a). The present study indicated that the expression of *PCaP2* in roots, cotyledons, true leaves, lateral roots and flowers was highly induced under cold stress, especially in roots (**Figure 1**), indicating that PCaP2 is widely expressed in response to chilling stress, suggesting that PCaP2 might play more important or multiple roles under cold stress. Previous studies have been found that PCaP2 functions in roots, such as root hair growth and abnormal root growth (Wang X. et al., 2007; Kato et al., 2013). Our data also showed that the expression of *PCaP2* was the highest in roots, compared with the other tissues, in cold and ABA treatments (**Figures 1**, **3B,C**), suggesting that the role of PCaP2 in response to chilling and ABA might mainly result from plant root responses. Further, we found that *PCaP2*-OE expressed the enhanced tolerance, while *PCaP2*-RNAi or *pcap2* were hypersensitive to chilling stress in seed germination, seedling and reproductive growth, and the difference of these phenotypes was dependent on the expression level of *PCaP2* (**Figure 2**), demonstrating that PCaP2 is an important and positive regulator of plant chilling tolerance.

Previous studies have proved that an elevated cytoplasmic Ca^2+^ level by environment signaling may trigger the interaction of CaM with PCaP2, resulting in PtdInsP2s becoming free and interacting with the target membrane proteins, demonstrating that PCaP2 is involved in intracellular signal transduction (Kato et al., 2010a,b, 2013). Our results showed that PCaP2 can regulate the expression of many ABA responsive-genes and cold inducible-genes, including some upstream genes such as *CBFs*, *SnRK2.2*, -*2.3* (**Figures 4**–**6**), suggesting that PCaP2 might mainly function as a Ca^2+^-binding protein to participate in intracellular signaling transduction under chilling stress.

Considering that both PCaP1 and PCaP2 also function in regulating MT destabilizing and F-actin severing (Wang X. et al., 2007; Zhu et al., 2013), the question is raised whether these functions of PCaP2 are also involved in plant chilling tolerance. Our results suggested that the F-actin-severing activity of PCaP2 wasn’t involved in response to chilling stress in seedling growth (Supplementary Figure S2), however, PCaP2 may be responsive to chilling stress partially by the dynamic organization of MTs (**Figure 1**). Although there are reports that a plant with increased chilling tolerance along with an elevated cold stability of MTs (Jian et al., 1989; Nick, 2013), our results indicated that it is possibility that PCaP2 functions as a MT destabilizing protein and enhances the ability of plant chilling tolerance and triggers cold- and ABA- responsive gene expression, suggesting MT destabilization also confers plant chilling tolerance, which is a novel phenomenon in MT-mediated plant cold responses. It has been proved that the high dynamics and reorganization of MT respond to environment signals and the early MT destabilization may improve the later MT stability in some environment conditions, such as cold acclimation (Wang and Nick, 2001; Abdrakhamanova et al., 2003), salt stress (Wang C. et al., 2007), thus we speculate that the MT destabilization in chilling stress might play a similar role. In addition, it is well-known that MTs can regulate the plant growth (Mathur, 2004; Wang X. et al., 2007), supporting that PCaP2 as MT-associated protein could be involved in the regulation of plant growth in chilling stress. However, it is hardly to control the steady environment conditions of low temperature in observing the dynamics of MTs in live-cells. Thus, we can’t obtain the results about the dynamics of MTs under cold stress.

### PCaP2 Is a Positive Regulator of ABA and a Linker Between Cold and ABA Signals by Activating CBF-COR Pathway

Transcriptional cascades, mediated by CBF regulators, are the central regulatory framework in a cold signaling pathway (Zarka et al., 2003; Cook et al., 2004; Gilmour et al., 2004). Our results proved that PCaP2 can trigger CBF-*COR* pathway in plant chilling tolerance due to the evidences that the mutant of *PCaP2* obviously inhibited the expression of *CBF1*, -*3* and CBF-target *COR* genes, while increased the expression of *CBF2* in cold stress (**Figure 4**).

To this day, although no phenotypes of the single mutants of *CBF1*, -*2*, -*3* are reported in Arabidopsis chilling tolerance possibly due to the redundant function of CBFs in cold stress (Park et al., 2015), Arabidopsis *CBF1* overexpression seedlings and Arabidopsis *CBF3* overexpressing cassava seedlings obviously improve chilling tolerance by genetics analysis (Yang et al., 2010; An et al., 2016), indicating that CBF1 and CBF3 act as positive regulators of chilling tolerance. Previous studies demonstrate that CBF2 is a negative regulator of freezing and negatively mediates the *CBF1* and *CBF3* expression in cold stress (Novillo et al., 2004), suggesting the involvement of CBF2 in plant cold tolerance is the contrary pathway of CBF1, -3. In addition, two reports showed the different chilling-inducible phenotypes of the triple mutants of *CBF1*, -*2*, -*3* in Arabidopsis. One is no obvious phenotype with 4°C treatments from 45 to 90 days (Zhao et al., 2016); the other is chilling tolerance treated with 4°C from 35 to 50 days (Jia et al., 2016). On basis of the previous results, a possible explanation is the opposite pathways of CBF1, CBF2, and CBF3 resulting in the triple mutants inhibiting the expression of *CBF1*, -*3* to decrease chilling tolerance and inhibiting the expression of *CBF2* to increase chilling tolerance at the same time, thus the triple mutants might be hard to show a steady phenotype in different cultivation environments, although all treated with 4°C (Jia et al., 2016). Our studies further support the possibility that PCaP2 may regulate CBF1/3 and CBF2 through different pathway in response to chilling stress (**Figure 4**). Interestingly, our results showed that PCaP2 significantly inhibited the expression of *CBF2*, -*3* and slightly inhibited the *CBF1* expression in normal condition (**Figure 4**), suggesting PCaP2 might directly regulate the expression of *CBF2*, -*3* under normal condition, while *CBF1* is specially regulated by PCaP2 in chilling stress.

C-repeat binding factor-mediated pathway has been considered in a cross talk with ABA signaling pathway (Shi and Yang, 2014). Genetic studies also indicate that ABA biosynthesis and signaling components are important for the expression of *COR* genes, such as *RD29A*, *RD22*, *KIN1*, *KIN2*, *COR15A*, *COR15B*, and *HAB1* (Xiong et al., 2001, 2002). A report show that CBF-*COR* pathway can be triggered by ABA signals in cold stress and be mediated by MYB96-HHP module (Lee and Seo, 2015), however, the function of MYB96-HHP by genetics analysis remains unknown in chilling, freezing or ABA treatments. Thus, the crosstalk mechanism between CBF-*COR* pathway and ABA pathway is largely unknown in chilling stress.

Compared with the previous studies, our results provided the more details. ABA upregulated the expression of *PCaP2* in roots, cotyledons, true leaves, and lateral roots (**Figures 3B,C**), and *PCaP2*-OE seedlings were less sensitive, while *PCaP2*-RNAi or *pcap2* seedlings were hypersensitive to exogenous ABA treatments, which was consistent with the phenotypes treated by chilling stress (**Figures 3E,F**). In addition, *pcap2* inhibited the expression of *CBF1*, -*3*, *RD29A*, *KIN1*, *KIN2*, *COR15A*, and *HAB1*, but increased the *CBF2* expression, in exogenous ABA treatments, which was similar expression change treated by cold (**Figure 4**). These results suggest that PCaP2 is a positive regulator of ABA signaling pathway and may activate the CBF-mediated transcriptional network by ABA signal in cold stress.

### SnRK2s Are Involved in Response to Cold Stress and Mediated by PCaP2

SnRK2s are a center of ABA signaling pathway (Kulik et al., 2011). Others have demonstrated that the transcription level of SnRK2s is upregulated in response to cold stress in some species. For example, the transcription level of TaSnRK2.4, TaSnRK2.7, TaSnRK2.8 (in wheat), ZmSnRK2.3, and ZmSnRK2.7 (in maize) (Zou et al., 2006; Huai et al., 2008; Zhang et al., 2010, 2011). However, the few investigations of Arabidopsis, SnRK2s have been conducted in cold stress. In Arabidopsis, SnRK2.2, -2.3, -2.6 are crucial in response to abiotic stress (Fujii and Zhu, 2009). Our results firstly proved the involvement of SnRK2.2, -2.3, -2.6 in cold stress by the analysis of the cold inducible expression of *SnRK2.2*, -*2.3*, -*2.6* and SnRK2-mediated genes (**Figure 6**). It has been proved the functional segregation of between SnRK2.6 and SnRK2.2, -2.3 in drought stress (Fujii et al., 2007; Kulik et al., 2011). Interestingly, our further studies showed that PCaP2 positively influenced both ABA-induced and cold-induced the expression of *SnRK2.2*, -*2.3*, but not *SnRK2.6* (**Figure 6**), demonstrating that PCaP2 triggers the expression of *SnRK2.2*, -*2.3* and the separately function of SnRK2.6 and SnRK2.2, -2.3 might be also involved in cold stress.

In ABA signaling pathway, SnRK2s play a key role in stimulating the expression of ABA-dependent transcription factors, such as ABIs and ABFs, which mediate the expression of many downstream ABA responsive genes (Fujii and Zhu, 2009). In Arabidopsis, four AREB/ABF transcription factors and five ABIs can be mediated by SnRK2s (Shi and Yang, 2014; Trivedi et al., 2016). Our results showed that PCaP2 regulated the expression of *ABF2*, *KIN1*, *KIN2*, and *RD29A* in ABA and cold treatments (**Figures 5**, **6**), illustrating that PCaP2 also increase the SnRK2-mediated gene expression in cold stress and ABA treatments, further supporting that PCaP2 activates the SnRK2-mediated transcriptional network in response to ABA and cold signals.

Among ABFs and ABIs, the expression of *ABF1* is significantly induced by cold (Choi et al., 2000; Uno et al., 2000) and ectopic expression *ABI3* confers ability to express *COR* genes and enhances cold tolerance in Arabidopsis (Tamminen et al., 2001), illustrating that ABF1 and ABI3 is involved in cold stress. One study illustrates that ABF/AREB family transcription factors physically interact with DREB/CBF family members, and ABF2 interacts with CBF3 *in vitro* (Lee et al., 2010), suggesting that ABF2 could function by associating with CBF-pathway. Our results showed that only ABF2 expression was positively mediated by PCaP2 in ABA and cold treatments (**Figures 5**, **6**), suggesting that the involvement of PCaP2 in chilling stress might be a same pathway with ABF2.

## Conclusion

PCaP2 positively regulates the seed germination, the seedling and reproductive growth, and activates CBF- and SnRK2-mediated transcriptional network in chilling stress and ABA response, highlighting that PCaP2 plays an important role in the crosstalk between chilling and ABA signals, providing novel evidences relating to underlying mechanisms of multi-pathways in plant chilling tolerance may allow us to gain further insights into how plants respond to chilling cues.

## Materials and Methods

### Plant Materials

Arabidopsis ecotype Col-0 was the background for all transgenic and mutant plants in this study. The seeds of *pcap2*, *PCaP2*-RNAi and *PCaP2*-OE, *m1*, *m2*, *m7* and p*PCaP2* promoter::GUS were provided by professor Ming Yuan and professor Ying Fu, China Agricultural University, Beijing, China. All these seeds were used in the previous studies (Wang X. et al., 2007; Kato et al., 2013; Zhu et al., 2013).

### Growth Conditions and Phenotype Analysis

Arabidopsis seeds were surface-sterilized and sowed on MS/G plates consisting of 1/2 MS (Murashige and Skoog) medium with vitamins (PlantMedia^1^), 0.6% w/v phytoagar (PlantMedia), and 1% w/v sucrose, pH adjusted to 5.8–6.0 with 1 M NaOH. The plates were cold-treated at 4°C in darkness for 3 days, and then moved to a growth chamber at 22°C, with a 16 h/8 h (light/dark) photoperiod at approximately 120 μmol m^-2^ s^-1^. For chilling tolerance assay, 4-day-old seedlings were transferred to new 1/2 MS medium and then placed in a growth chamber set at 14°C while the control group set at 22°C for 11 days (**Figures 2B,C**), additionally, 7-day-old seedlings which grown at normal condition were transferred to matrix soil at 22°C for 40 days (the top of **Figure 2D**) or 14°C for 60 days (the bottom of **Figure 2D**), respectively. For ABA treatment, 4-day-old seedlings were transferred to 1/2 MS medium without or with ABA (0.5 μM). The growth phenotypes including leaf numbers, leaf area, primary root length, and lateral root numbers of chilling stress and ABA treatments were investigated and photographed. At least 20 seedlings in each experiment were observed and the experiments were repeated at least three times.

### RNA Isolation and Quantitative RT-PCR (qRT-PCR)

Wild type and *PCaP2*-OE seedlings grown for 14-day-old at 22°C were harvested. Total RNA was isolated using an RNeasy Plant Mini Kit (Qiagen), and treated with RNase-Free DNase (Qiagen) to remove DNA contamination by the manufacturer’s instructions. cDNA was synthesized using 1 μg total RNA by oligo- (dT) 20-primed reverse transcription using the Omniscript RT Kit (Qiagen). The total reaction volume was 20 and 1 μL of the reaction mixture was subjected to the quantitative real time PCR reaction. *18S rRNA* was used as an internal control, the expression levels of *PCaP2* were analyzed. Three biological replicates and two to three technical replicates (for each biological replicate) were used for each treatment. The average and standard deviation were calculated from the biological replicates.

To assay the expression of *PCaP2* under chilling stress, 14-day-old or 4-week-old WT seedlings grown at normal condition were incubated at 4°C for 0, 1, 3, 6 and 12 h. The 14-day-old seedlings were used for detecting the expression level of *PCaP2* in various issues while 4-week-old seedlings were used for flower tissues. WT, *pcap2* and *PCaP2*-OE seedlings grown for 14-day-old at normal condition were incubated at 4°C for 0 or 12 h used for analyzing the expression of chilling response genes. To assay the expression levels of *PCaP2* after ABA treatment, qRT–PCR analysis was performed with RNA samples isolated from 14-day-old WT seedlings harvested at 0, 1, 3, 6, and 12 h after treatment with 40 μM ABA. To assay the expression of ABA response genes in WT and mutant lines by ABA treatment, WT and mutant seedlings grown at normal condition for 14-day-old were harvested and treated with 40 μM ABA for 0 and 12 h. Total RNA extraction and reverse transcription were performed as described above. All the primers used for qRT-PCR examination are listed in Supplementary Table S1. Three replicates were performed for each representative experiment.

### Histochemical Staining of GUS Activity

Fourteen-day-old p*PCaP2* promoter::GUS seedlings were collected and incubated at 4°C for 6 h or 40 μM ABA for 3 h, then used for observing the changes of GUS activity under chilling stress and ABA treatment in various tissues. The GUS staining procedure was performed by the method from Wang X. et al. (2007). Samples were examined on an Olympus microscope equipped with a color CCD camera (Sutter Instrument; LAMBDA 10-2) or by an Epson scanner.

## Author Contributions

CW and XW designed the study. LW, YW, and HL performed the experiments and data analysis. DH and DS provided help in experimental methods. SZ, HC, NZ, and QC participated in the discussion. ST and ZZ trained the use of experimental equipment. CW and XW wrote the manuscript.

## Conflict of Interest Statement

The authors declare that the research was conducted in the absence of any commercial or financial relationships that could be construed as a potential conflict of interest.

## Footnotes

1 www.plantmedia.com

## Abbreviations

ABA: abscisic acid
ABI: abscisic acid insensitive
AREBs/ABFs: ABRE-binding proteins/factors
Ca^2+^: calcium ion
CaM: calmodulin
CBF/DREB1: C-Repeat Binding Factor/DRE Binding Factor1
COR: cold-responsive
F-actin: filamentous actin
GA3: gibberellin3
GUS: β-glucuronidase
MS: Murashige and Skoog
MT: microtubule
PCaP2: plasma membrane-associated Ca^2+^-binding protein-2
*PCaP2*-OE: PCaP2 overexpression
PCaP2-RNAi: *PCaP2* RNA interference
qRT-PCR: quantitative real-time PCR
ROS: reactive oxygen species
RT-PCR: reverse transcriptase-mediated PCR
SA: salicylic acid
WT: wild type.
