# Supplementary material for: Arabidopsis PCaP2 Plays an Important Role in Chilling Tolerance and ABA Response by Activating CBF- and SnRK2-Mediated Transcriptional Regulatory Network
Source: Front Plant Sci. 2018 Mar 8;9:215. doi: 10.3389/fpls.2018.00215 (PMC5852069; doi:10.3389/fpls.2018.00215)
Supplement: Supplementary file 1 [file Table_1.DOC]

Supplementary Table S1 Primers used in this study

| Gene name | Primer sequence (5’ to 3’) |
| --- | --- |
| *SnRK2.2* | Forward: TGGAATATGCTGCTGGTGGAGAACTTTATGAG |
|  | Reverse: TGGTTGGGAATGAAGAACAGAAGACTTGAGAG |
| *SnRK2.3* | Forward: TCTGGCGGTGAACTTTACGAGCGGATTTG |
|  | Reverse: CCTGACGAAGCAGTACCTCTGGAGCGATG |
| *SnRK2.6* | Forward: CACAGAGATTGAAGTCGCAAAGAGACAGAG |
|  | Reverse: CGGAGCCAATATCCTTGACGAGTTCATACC |
| *ABF1* | Forward: GGAGAAGGTTGTTGAGAGAAGG |
|  | Reverse: AGCTTCCAGTTCCAAGGTATAAG |
| *ABF2* | Forward: CACAGCACCAACGCCTAAAGC |
|  | Reverse: ATCGCAACAGCAACAGCCAATC |
| *ABF3* | Forward: ATCGTCCGAGGCAAGGTAAGTG |
|  | Reverse: TGATGGTGTGAGTGAGCAGCAG |
| *ABF4* | Forward: ATCTGCTGTTGTTGCTGCTGAAG |
|  | Reverse: ACTGCTGCTGGCGGCTTAG |
| *RD29A* | Forward: GTCTCCGTCTTTGGGTCTCTTCC |
|  | Reverse: TTCTCCGATGGGCTTTGGTAGTG |
| *KIN1* | Forward: GGAAGGCATTCTTGTTGGTCTCTG |
|  | Reverse: GCCCACATCTCTTCTCATCATCAC |
| *KIN2* | Forward: ACTCGGATCGCTACTTGTTCAGG |
|  | Reverse: AGGCGGGAAAGAGTATATCGGATG |
| *COR15B* | Forward: CAGTGGCATGGGTTCTTCTTTCC |
|  | Reverse: TTTCTTTGTGGCTTCGTTGAGGTC |
| *18s rRNA* | Forward: CGGCTACCACATCCAAGGAA |
|  | Reverse: GCTGGAATTACCGCGGCT |
| *PCaP2* | Forward: CCAGCCGTAGAAGAGGAGAAGAAG |
|  | Reverse: GGAGTTTCGGGAGCCTTAGTCG |
| *CBF1* | Forward: TTCTCCGATGGGCTTTGGTAGTG |
|  | Reverse: GTCTCCGTCTTTGGGTCTCTTCC |
| *CBF2* | Forward: AGAATGGCGGCGGGCAAAG |
|  | Reverse: GCGTCTCCTTCACTCCACTTCC |
| *CBF3* | Forward: TCTTACTGCTGAAGGTTCGTCTGG |
|  | Reverse: CGAGGTACTGGTGGTCTGTGC |
