# Supplementary figures and images for: Arabidopsis PCaP2 Plays an Important Role in Chilling Tolerance and ABA Response by Activating CBF- and SnRK2-Mediated Transcriptional Regulatory Network
Source: Front Plant Sci. 2018 Mar 8;9:215. doi: 10.3389/fpls.2018.00215 (PMC5852069; doi:10.3389/fpls.2018.00215)

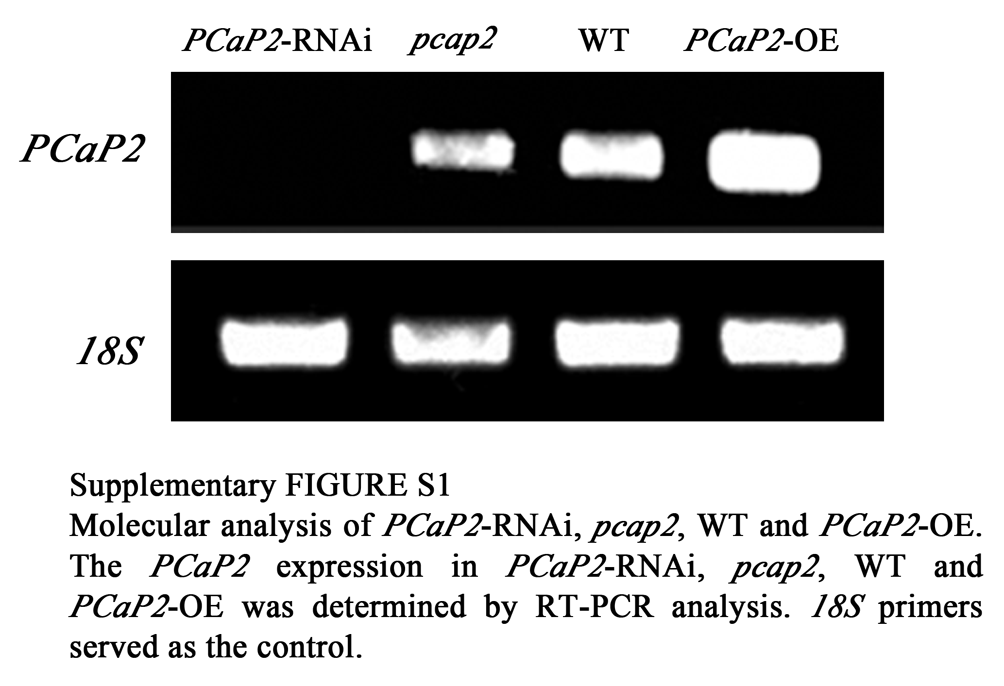

Supplement: Supplementary file 2 [file Image_1.TIF]

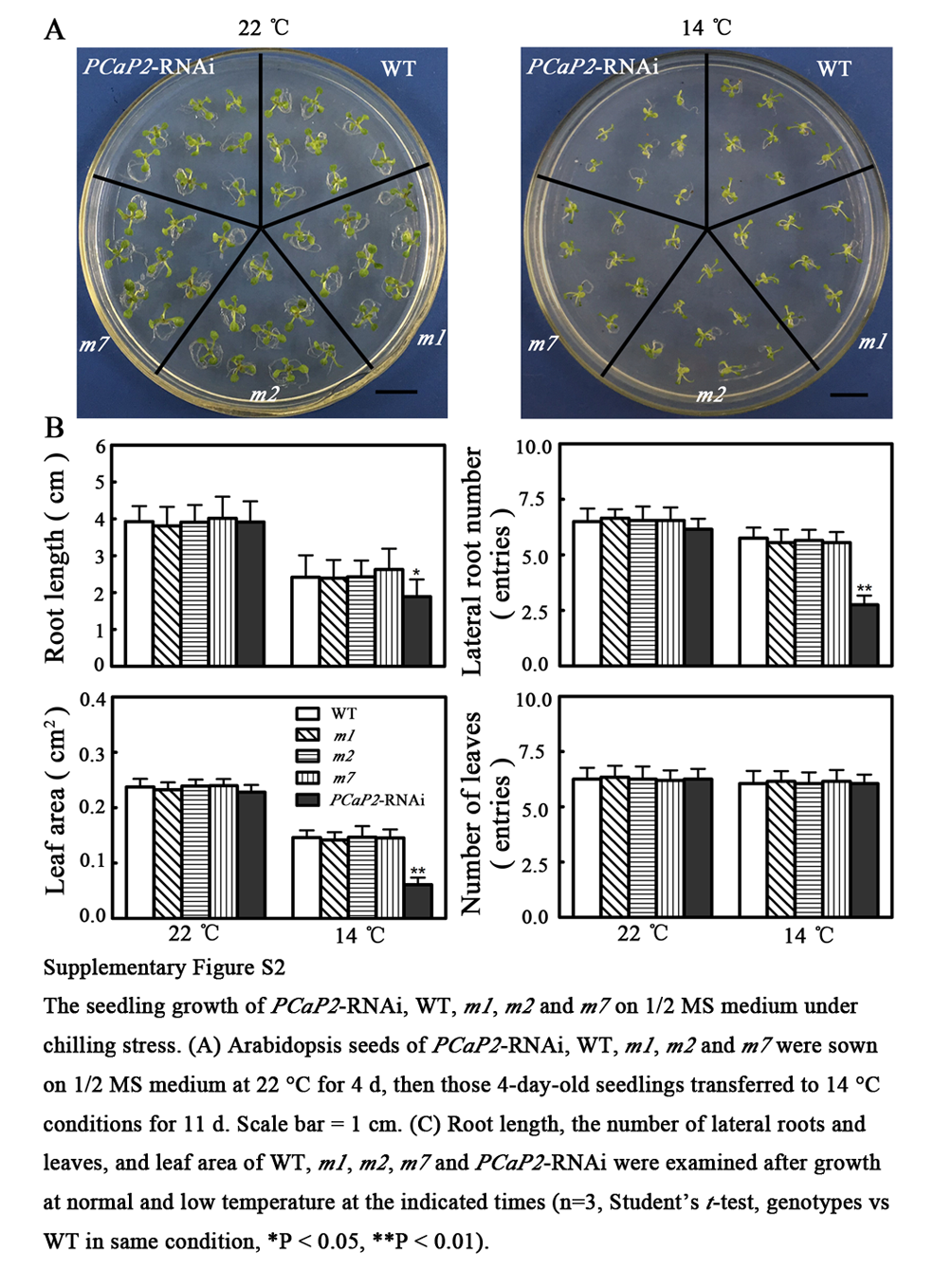

Supplement: Supplementary file 3 [file Image_2.TIF]
